# Ground Calcium Carbonate as a Low Cost and Biosafety Excipient for Solubility and Dissolution Improvement of Praziquantel

**DOI:** 10.3390/pharmaceutics11100533

**Published:** 2019-10-14

**Authors:** Ana Borrego-Sánchez, Rita Sánchez-Espejo, Beatrice Albertini, Nadia Passerini, Pilar Cerezo, César Viseras, C. Ignacio Sainz-Díaz

**Affiliations:** 1 Instituto Andaluz de Ciencias de la Tierra (CSIC-University of Granada), Av. de las Palmeras 4, 18100 Granada, Spain; ritaespejo@hotmail.com (R.S.-E.); cviseras@ugr.es (C.V.); ignacio.sainz@iact.ugr-csic.es (C.I.S.-D.); 2 Department of Pharmacy and Pharmaceutical Technology, Faculty of Pharmacy, University of Granada, Campus de Cartuja s/n, 18071 Granada, Spain; mcerezo@ugr.es; 3 Department of Pharmacy and BioTechnology, University of Bologna, Via S. Donato 19/2, 40127 Bologna, Italy; beatrice.albertini@unibo.it (B.A.); nadia.passerini@unibo.it (N.P.)

**Keywords:** praziquantel, calcium carbonate, schistosomiasis, bioavailability, solubility, cytotoxicity

## Abstract

Calcium carbonate is an abundant mineral with several advantages to be a successful carrier to improve oral bioavailability of poorly water-soluble drugs, such as praziquantel. Praziquantel is an antiparasitic drug classified in group II of the Biopharmaceutical Classification System hence characterized by high-permeability and low-solubility. Therefore, the dissolution rate is the limiting factor for the gastrointestinal absorption that contributes to the low bioavailability. Consequently, the therapeutic dose of the praziquantel must be high and big tablets and capsules are required, which are difficult to swallow, especially for pediatric and elderly patients. Mixtures of praziquantel and calcium carbonate using solid-solid physical mixtures and solid dispersions were prepared and characterized using several techniques (X-ray diffraction differential scanning calorimetry, thermogravimetric analysis, scanning electron microscopy, laser diffraction, Fourier transform infrared and Raman spectroscopies). Solubility of these formulations evidenced that the solubility of praziquantel-calcium carbonate interaction product increased in physiological media. *In vitro* dissolution tests showed that the interaction product increased the dissolution rate of the drug in acidic medium. Theoretical models were studied to understand this experimental behavior. Cytotoxicity and cell cycle studies were performed, showing that praziquantel-calcium carbonate physical mixture and interaction product were biocompatible with the HTC116 cells, because it did not produce a decrease in cell viability or alterations in the cell cycle.

## 1. Introduction

Calcium carbonate, CaCO_3_, being a low cost material, with high surface area, excellent safety, biocompatibility and biodegradability, is a well-documented excipient in pharmaceutical solid dosage forms, mainly used as diluent [1]. Recently, it has also been demonstrated that calcium carbonate can successfully act as hydrophilic porous carrier to improve the oral bioavailability of low water soluble drugs [2,3,4,5]. Functionalization with hydroxypropyl-β-cyclodextrin [6], enzymatic macromolecules [7], or incorporation in polymeric hydrogels [8,9,10,11] can further enhance the ability of calcium carbonate to improve oral delivery of several biomolecules, including proteins [12] and anti-cancer drugs [13]. As recently reviewed, most of the successfully designed calcium carbonate carriers have been prepared by emulsion techniques or chemical precipitation of the carbonate micro/nanoparticles [14]. However, precipitation of calcium carbonate micro/nanoparticles is difficult to reproduce and scale up of the procedure is most of times challenging, avoiding the clinical use of these carriers. Moreover, detailed understanding of biosafety and *in vivo* degradation of the new calcium carbonate particles would require preclinical studies. An interesting alternative is to overcome these challenges by using normalized pharmaceutical excipients grades of calcium carbonate as drug delivery carriers [1].

Praziquantel (PZQ) is the drug of choice in the treatment of schistosomiasis [15], being included in the WHO Model List of Essential Drug for the treatment of adults and children [16]. Schistosomiasis affects approximately 210 million people, causing 200,000 deaths every year. Moreover, it is widely extended, mainly in 78 developing countries in the tropics and subtropics, although at least 92% of people who need treatment for schistosomiasis live in Africa and, is the second of the most prevalent disease (after malaria) affecting African children. PZQ is classified in group II of the Biopharmaceutical Classification System (BCS) and hence characterized by high permeability and low solubility [17]. Therefore, the dissolution rate is the limiting factor for the gastrointestinal absorption that contributes to the low oral bioavailability. Moreover, absorbed amounts of PZQ suffer an extensive first-pass metabolism [18], leading to administration of high-dosed dosage forms, which are difficult to swallow, especially for pediatric patients [19].

Enhancing the water solubility and the dissolution rate is critical to increase the oral bioavailability of PZQ. Several studies and strategies have been carried out to improve PZQ water solubility, such as incorporation into liposome vectors [20], preparation of solid dispersions of the drug with different excipients such as clay minerals [21], β-cyclodextrins [22,23,24], polyvinyl-pyrrolidone [25,26,27], polyethylene glycols [28,29,30], sodium starch glycolate [31] and preparation of dispersible granules [14]. In addition, other techniques have been used to increase the solubility of PZQ such as co-grinding with several excipients [32], melt granulation and ultrasonic spray congealing [27], and the transition in a new crystalline polymorph by milling [19]. More recently, our previous paper evidenced conformational changes of PZQ in association with calcium carbonate after a solvent evaporation process [33]. This interaction product revealed the formation of modifications of the pristine racemic drug attributable to either polymorph B [19] or disordered pseudoracemate solid phases [33].

Given these premises, the first aim of this work was to verify if PZQ solid state modification within calcium carbonate solid dispersion is due to the solvent evaporation process or to drug-carrier interactions that would happen during a mere blending procedure. Thus, the physico-chemical properties of the physical mixture were compared to those of the interaction product. Further, we explored the possibilities of calcium carbonate/PZQ systems for the improvement of biopharmaceutical properties of the drug. In particular, solubility and dissolution profiles of PZQ from interaction product were compared to the corresponding physical mixture, and the results explained by using theoretical modeling. In addition, we investigated the *in vitro* cytotoxicity and cell cycle studies of calcium carbonate/PZQ systems.

## 2. Materials and Methods

### 2.1. Materials

PZQ drug was kindly donated by Fatro S.p.A. (Bologna, Italy). Ground Calcium Carbonate Calcitec Pure PH V/40S (GCC) was purchased from Mineraria Sacilese (Sacile, Italy). This kind of calcium carbonate is used in the pharmaceutical industry and was accepted in the latest edition of the European and United States Pharmacopoeias. The GCC finds also employment in the food industry and in industries where is requested a low content of heavy metals. Ethanol of 96% of purity was used as solvent.

### 2.2. Preparation of the PZQ and GCC Physical Mixture

Physical mixtures (PM) of PZQ and GCC (1:5 *w/w*) were prepared blending both solids in an agate mortar of 100 mm of diameter at room temperature for 5 min.

### 2.3. Preparation of the PZQ and GCC Interaction Product (IP)

GCC was dispersed in 1 L of ethanolic solution of PZQ under magnetic stirring at room temperature for 24 h, so drug/GCC ratio was 1:5 *w/w* in order to ensure the complete interaction between PZQ and GCC. After 24 h, the solvent was evaporated with rotary evaporator (Rotary evaporator Buchi^®^ R II, Flawil, Switzerland) at 40 °C and reduced pressure. The solid residue was dried in a desiccator and then it was pulverized.

### 2.4. Solid State Characterization of Calcium Carbonate/Praziquantel Systems

#### 2.4.1. X-ray Diffraction (XRD)

An X-Pert Pro^®^ diffractometer (Marvel Panalytical, Madrid, Spain) with the CuKα radiation was used for performing powder X-ray diffraction. The powder samples were scanned in the range of 4°–70° of the 2θ angle, steps were of 0.008 of 2θ and the counting time was of 10.16 sec/step. The diffraction results were analyzed with the XPOWDER^®^ software version 2004 [34].

#### 2.4.2. Thermal Analysis

Differential scanning calorimetric analysis (DSC) and thermogravimetric analysis (TGA) were performed with a mod. TGA/DSC1 calorimeter (Mettler Toledo, Barcelona, Spain) equipped with a sensor and FRS5 microbalance (precision 0.1 μg) and FP89 software package. Samples were heated in air atmosphere at 5 °C/min in the in the 30–200 °C temperature range for DSC and 30–420 °C temperature range for TGA.

#### 2.4.3. Scanning Electron Microscope (SEM)

Microphotographs of the samples were performed using a Hitachi S-510 scanning electron microscope (voltage 25 kV, secondary electron images) (Hitachi Scientific Instruments Ltd., Tokyo, Japan). The samples were mounted on adhesive carbon paper, fixed with colloidal gold and metallized with gold in two orientations (20–30°). The images were captured digitally using the program attached to the microscope (ScanVision, Version 1.2).

#### 2.4.4. Particle Size Analysis

The particle size distribution of the solid sample materials suspended in liquid medium was analyzed with Laser Light Diffraction technology. The equipment used was a Mastersizer 2000LF from Malvern Panalytical Instruments (Madrid, Spain) consisting of HYDRO MU Malvern manual liquid sample dispersion unit and Malvern HYDRO 2000Up minimum volume liquid sample dispersion unit.

#### 2.4.5. Fourier Transform Infrared and Raman Spectroscopies

Fourier Transform Infrared Spectroscopy (FTIR) spectra were recorded with a 6200 spectrophotometer (JASCO, Pfungstadt, Germany) in the range 4000–600 cm^−1^ with a 0.5 cm^−1^ resolution and a well-plate sampler along with the Spectra Manager II software.

Raman spectra were recorded using a JASCO NRS-5100 Micro-Raman dispersive spectrophotometer in the range 3500–800 cm^−1^ with a 6.48 cm^−1^ resolution, with laser light source VIS-NIR with red diode at 785 nm with 500 mW of power (Torsana Starbright) refrigerated by air and the KnowItAII JASCO for Raman software.

### 2.5. Solubility Studies

The solubility of PZQ as well as the physical mixture PM PZQ-GCC and the interaction product IP PZQ-GCC were studied separately in two media: an acidic medium of HCl 0.001 M and a simulated intestinal fluid (SIF) without enzymes with a buffer at pH 6.8. The solubility of PZQ was calculated by placing a supersaturated solution of the pristine drug, specifically 30 mg in 10 mL in each of the media. The supersaturated solution was stirred in a thermostatic bath for 72 h at 37 °C. After 72 h, it was centrifuged, and the supernatant was filtered and measured on high-performance liquid chromatography (HPLC). Obtaining by means of the HPLC the amount of dissolved drug that corresponds to its solubility. This experiment was repeated 6 times. In the same way this procedure was applied for the PM and the IP PZQ-GCC.

### 2.6. Dissolution Studies

PZQ-GCC physical mixture or PZQ-GCC interaction product (210 mg) were encapsulated in double zero (00) gelatin capsules, corresponding to 35 mg of PZQ and 175 mg of GCC in each capsule. As well as, 35 mg of PZQ were encapsulated as reference. The obtained capsules were subjected to sink conditions dissolution tests using the official Pharmacopoeia USP apparatus 2 for the dissolution test of oral solid dosage forms (Sotax AT7, S). This apparatus is equipped with a rotation system type palettes and sinkers, piston pump for the automatic sampling at scheduled times and collector of fractions. The measurements were performed at 37 °C and 150 rpm in 1 L of medium. Two separate dissolution media were studied: an acidic medium of HCl 0.001 M (simulated stomach) and a SIF medium without enzymes with a buffer at pH 6.8 (simulated intestine). Aliquots of 5 mL were collected from the dissolution test, filtered through 0.45 μm Millipore^®^ (S) membranes and analyzed by HPLC for drug content. Volumes of 5 mL of fresh dissolution medium were replaced after each sampling to maintain the volume constant. At least three replicates for each sample were assayed.

### 2.7. HPLC Analysis

Drug analysis was performed using a 1260 Infinity II Agilent HPLC system (Santa Clara, CA, USA) equipped with quaternary pump, autosampler, column oven and UV-VIS diode-array spectrophotometer. The stationary phase was a Kromasil^®^ C18 column, 5 μm, 250 × 4.6 mm (Teknokroma, Barcelona, Spain) and the mobile phase was a mixture of H_2_O and CH_3_CN (35:65 *v/v*). The flow rate was set at 0.8 mL/min with an injection volume of 10 μL. A spectrophotometer detector at a 225 nm wavelength was used and the run time for each analysis was 5 min. Data were recorded and analyzed by using software LC Open LAB HPLC 1260 (Agilent). The response of the analytical method was linear in the concentration range 5–100 mg/L of PZQ in both media, resulting in correlation coefficients of 1 (both in HCl 0.001 M and SIF).

### 2.8. Computational Methods

The molecular model of PZQ was extracted from previous calculations [35] and the rest of components were placed by hand. The Compass force field (FF) [36] based on empirical interatomic potentials was used within the Discover program of the Materials Studio package [37]. This FF was used previously to describe PZQ molecules and crystal structure with satisfactory results [35,38]. An atomic interactions cut-off of 18.5 Å was used for calculating Van der Waals and Coulomb interactions.

### 2.9. Cell Culture

Cell viability tests and study the cell cycle profiles were performed for observing a possible production of cell death in a tumor line of cells derived from colorectal carcinoma, called HCT116. This tumor line of HCT116 colorectal carcinoma cells were cultured in Dulbecco’s Modified Eagle’s Medium (DMEM) (Gibco Invitrogen, Dublin, Ireland) supplemented 10% with decomplemented Heat-Inactivated Fetal Bovine Serum (FBS) (Gibco Invitrogen), with glutamax (BioWhittaker, Cologne, Germany) at 1% and with Penicillin/Streptomycin (BioWhittaker) at 1%. The cell culture was kept in an incubator at 37 °C and 5% of CO_2_.

### 2.10. Cytotoxicity Studies

Firstly, PZQ and GCC samples were prepared relying on the administered drug amount in animals as antiparasitic. In such way, the amount of the mineral was five times more than that of PZQ. To do this, we prepared an intermediate dilution in dimethylsulfoxide (DMSO) at a concentration of 100 mM and then another dilution at 10 mM. The physical mixture and the interaction product PZQ-GCC were studied using the same procedure.

To perform the proliferation assays by Alamar Blue, about 10,000–20,000 cells/well of HCT116 were seeded in 96-well plates, in a final volume of 200 μL of DMEM medium using several concentrations (100 μM, 20 μM, 4 μM, 800 nM, 160 nM, 32 nM and 6.4 nM) for each sample studied (PZQ, GCC, PM and IP PZQ-GCC). Cells were incubated at 37 °C, 5% CO_2_ for 48 h. After the incubation time, 10 μL per well of the PrestoBlue cell Viability Reagent (Invitrogen, Thermo Fisher Scientific, Carlsbad, CA, USA) was added, incubating for 15 min. After this period, fluorescence was measured at 535–90 nm in a Tecan reader (Männedorf, Switzerland). This reagent is based on resazurin compound, which works as an indicator of viability when it is reduced by living cells and presents a colour change from blue (dead cells) to pink (living cells).

### 2.11. Cell Cycle Studies

The cells were cultured in 24 well plates at a concentration of 250,000 cells/well in 500 μL and treated with the previously selected PZQ, GCC, PM and IP PZQ-GCC samples, at increasing concentrations of 0.8, 4, 20 and 100 μM for 48 h. Dead cells (apoptotic and necrotic) were detected based on staining with propidium iodide following the protocol described [39]. Briefly, the cells after the corresponding treatments were collected and washed with 2 mL of phosphate buffered saline (PBS) at 4 °C and fixed with 100 μL of PBS and 900 μL of 70% ethanol on ice for 5 min. After washing with PBS, they were resuspended in 250 μL of PBS and another 250 μL of a DNA extraction solution (0.2 M Na_2_HPO_4_, 0.1M C6H8O7, pH 7.8) and incubated at 37 °C for 10 min. The supernatant was removed and 200 μL of the staining solution was added (8 μL propidium iodide (1 mg/mL) and 2 μL RNAse 100 (μg/mL)), incubating the samples for 10 min at 37 °C in the dark. Fluorescence was measured in the FL2 detector of the FACScalibur cytometer (Becton Dickinson & Co., Franklin Lakes, NJ, USA) and the analysis of the sub-G1 population (population of dead cells: necrotic and apoptotic) was done using the BD CellQuest software v1.0.2 (BD, Biosciences).

## 3. Results and Discussion

### 3.1. Solid State Characterization of Calcium Carbonate/Praziquantel Systems

PZQ and GCC (1:5) solid dispersions obtained by solvent evaporation evidenced conformational changes of the pristine drug solid state [33]. Recently, a number of papers [19,32,40] have elucidated that the mechanochemical activation of PZQ, via neat grinding or comilling PZQ with different polymers (Povidone, Copovidone, Crospovidone and Sodium Starch Glycolate) enabled the transformation of the original PZQ polymorphic form into a new polymorphic variety of racemic PZQ or into a drug amorphous state.

In this study, the properties of PZQ-GCC physical mixture (produced by blending) were compared to those of the PZQ-GCC interaction product (obtained by solvent evaporation). A solid state characterization of the samples studied was performed by X-ray diffraction (Figure S1), differential scanning calorimetric analysis (Figure S2), thermogravimetric analysis (Figure S3), scanning electron microscope (Figure S4), Fourier Transform Infrared and Raman Spectroscopies (Figure S5) and particle size analysis. The results demonstrated that PZQ-GCC physical mixture did not show any chemical-physical change with respect to the pure drug. On the contrary, in the PZQ-GCC interaction product multiple changes were observed, as already described in a previous work [33]. The detailed results and analyses of the solid state characterization of the samples studied (PZQ, GCC, PM and IP) are provided in the Supplementary Material.

### 3.2. Solubility Studies

The solubility tests showed that the IP PZQ-GCC increased the solubility of the pure drug from 0.50 mg/mL to 1.42 mg/mL in an acid aqueous medium with 0.001 M HCl (pH = 3), due to its interaction with the GCC and the solubility of the GCC in acidic medium. On the contrary, the PM PZQ-GCC showed a small and unimportant increase in the solubility of the drug (Table 1). The results in a SIF medium (pH = 6.8) revealed that the IP PZQ-GCC increased the solubility of PZQ from 0.45 mg/mL to 0.73 mg/mL; while the PM showed a slight increase in the solubility of PZQ from 0.45 mg/mL to 0.50 mg/mL, which was not considered notable. Therefore, the solubility of the IP PZQ-GCC improves in acid and SIF media with respect to the PZQ and PM. This increase is much higher at pH 3 (Table 1).

### 3.3. Dissolution Studies

**Figure 1:**
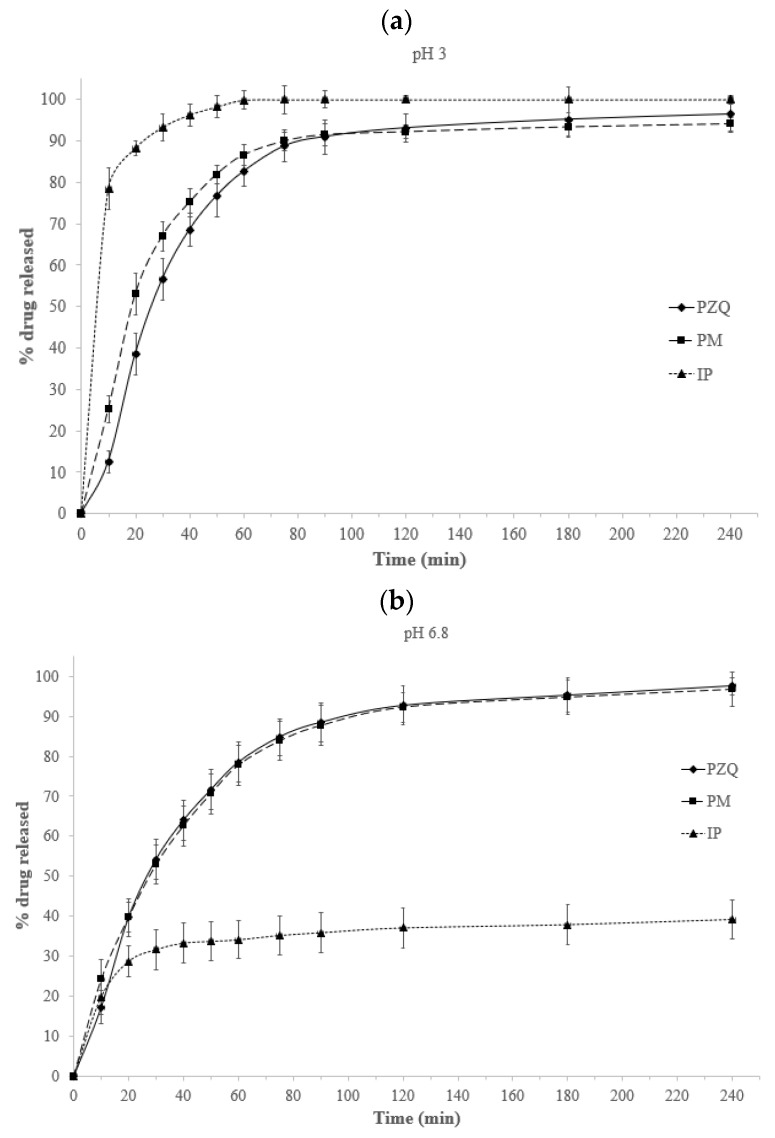
Drug release (% *w/w*) profiles from the PZQ and the PM in 0.001 M HCl at pH = 3 (**a**) and in a SIF medium at pH = 6.8 (**b**) in sink conditions; (mean values ± standard deviation; *n* = 5).

In order to explore the *in vitro* bioavailability of PZQ, dissolution tests in both media simulating the gastric and the intestinal fluids, were performed for the pristine drug and the combinations of PZQ with GCC (IP and PM) (Figure 1).

PZQ-GCC physical mixture presented a similar behavior to the pristine PZQ in both dissolution media (Figure 1a,b), while the IP showed a completely different behavior at pH 3 with respect to pH 6.8. Interaction product PZQ-GCC showed an increase in the dissolution rate in acid medium (Figure 1a) and a strong decrease in SIF (Figure 1b). These results were concordant with solubility tests results, where the solubility of IP PZQ-GCC is higher in acid medium than in SIF. This behaviour may be correlated to the different solubility of GCC at acidic and neutral pH and to structural changes observed in IP: as GCC is soluble at low pH, its interaction at molecular level with water and PZQ favoured the dissolution and solubility of the drug. Likewise, the PZQ-GCC interaction at pH 6.8, where the carbonate is completely insoluble, negatively affected the solubilisation of the PZQ molecules.

### 3.4. Modeling Approach

**Figure 2:**
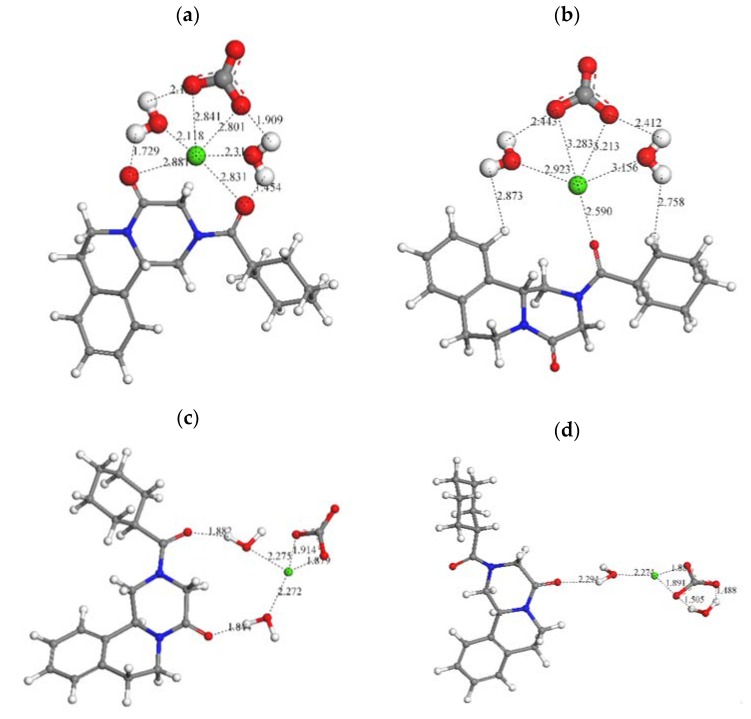
Possible complexes of PZQ molecule conformer *syn* (**a**) and *anti* (**b**) with hydrated Ca^2+^ carbonates clusters, and optimized complexes of *syn* (**c**) and *anti* (**d**) PZQ (non-bonding distances are included in Å). The C, Ca, H, N, and O atoms are in grey, green, white, blue, and red color.

In order to explain the dissolution behavior of the PZQ-GCC systems, small models of PZQ molecules with the GCC and water molecules were created (Figure 2). In the PM, the carbonyl groups of PZQ are at the same side of the molecule as a *syn* conformer, whereas in IP the proportion of the *anti* conformer is higher [38]. The geometry of these models was optimized with Compass FF calculations, using the SPC water model (Figure 2c,d). In both models the carbonate anion is coordinated with the Ca^2+^ cation *d*(Ca…OCO) = 1.88–1.91 Å and at least one water molecule is between the Ca^2+^ cation and a carbonyl group forming a hydrogen bond with the corresponding carbonyl group *d*(HOH…O=C) = 1.84–1.88 Å. In the *syn* conformer, both water molecules are coordinating the Ca^2+^ cation and the water H atoms *d*(HOH…Ca) = 2.27 Å (Figure 2c).

In the *anti* conformer, one water molecule is coordinating the Ca^2+^ cation, d(O…Ca) = 2.27 Å, and one carbonyl O atom d(CO…H) = 2.22 Å leaving the another carbonyl group free in the opposite side of the molecule to form an additional carbonate complex. The conformer *syn* can form only one complex with the hydrated Ca^2+^ cation (Figure 2). This could explain the higher dissolution process of the *anti* form of PZQ and hence the higher solubility of IP PZQ-GCC.

### 3.5. Cytotoxicity Studies

**Figure 3:**
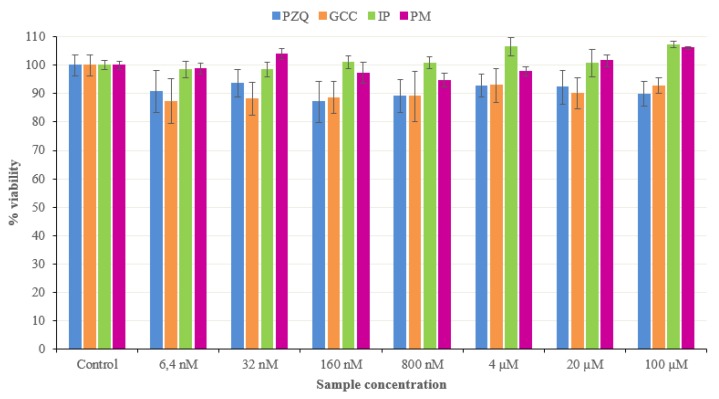
Cell viability from the studied samples after 48 h of treatment. (Control: untreated cells in complete medium; mean values ± standard error; *n* = 8).

*In vitro* cytotoxicity tests in the HCT116 cell line showed that all the PZQ and GCC pure concentrations (6.4 nM–100 μM) tested provided viability values slightly lower than the untreated cells (control). Therefore, PZQ and GCC pure samples can be considered biocompatible toward HCT116 cell lines (Figure 3). In the IP and PM PZQ-GCC products similar results were found, whose biocompatibility across the range of concentrations was evaluated and a trend similar to the control was observed and slightly positive with respect to the pure samples (PZQ and GCC) in cell growth. Therefore, it is notable that IP and PM PZQ-GCC products have a biocompatible behaviour, so the interaction between PZQ and GCC had a positive effect in the cell viability (Figure 3).

### 3.6. Cell Cycle Studies

**Figure 4:**
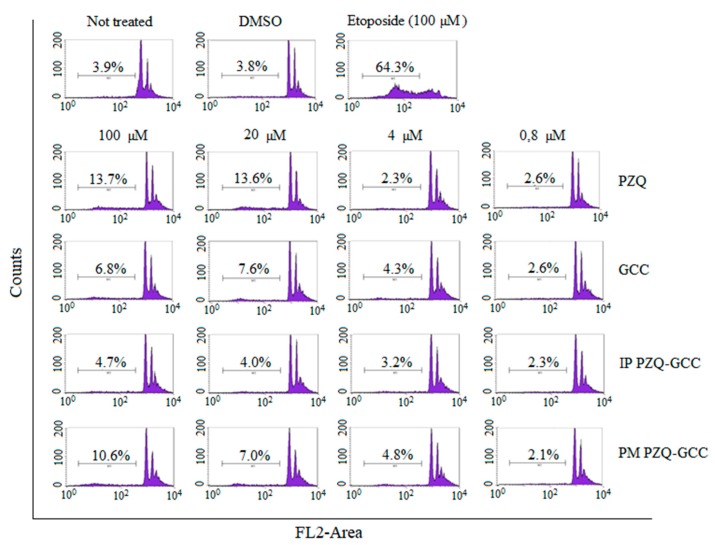
Cell cycle of the HCT116 line treated with the sample studies. The percentages indicate the number of cells in Sub-G1 (apoptotic or necrotic).

A study of the cell cycle by means of propidium iodide was carried out to study the cell cycle corresponding to the Sub-G1 phase of the cells in contact with our samples (Figure 4). Once observed through cytotoxicity studies that the samples tested did not cause cell death, a cell cycle study was carried out to check whether the compounds affected any phase of the cell cycle despite not affecting proliferation. In the upper part of the figure the control tests can be observed: an example of the cell cycle of healthy untreated cells, an example of cells treated with DMSO, where it is observed that DMSO does not affect the cell and also an example of cells treated with etoposide, an antineoplastic drug that damages cells. In this last example, the cell cycle of the control cells is severely affected and induces death to 64.3% of the cell population.

The studied samples showed that they do not affect in any case the cellular cycle of the cells, only in some cases at high concentrations can the cell death increase slightly, although not in a noteworthy way (Figure 4).

## 4. Conclusions

Interaction products obtained with PZQ and GCC increased the solubility of PZQ in physiological media, particularly in acid medium. *In vitro* dissolution tests evidenced that the interaction product increased the dissolution rate of the drug in acidic medium. *In vitro* cytotoxicity and cell cycle studies were performed, showing neither the PZQ-GCC physical mixture nor the interaction product produced cellular damage in any of the studied concentrations. Therefore, the samples studied did not produce cell death or alteration in the cell cycle and were biocompatible with the HTC116 cells.

As a general conclusion, the use of low cost GCCimprove solubility and dissolution rates of PZQ, without producing cytotoxicity or alterations in the cell cycle of HTC116 cells. This allows exploring the use of GCC as an interesting technological strategy for a drug administration in a more effective biopharmaceutical and clinical way ofPZQ. Also providing the great advantage that the design and development of the PZQ-GCC new formulations would not increase the final cost of the drug, overcoming a great challenge due to the population which the treatment is destined and the high prevalent of the neglected tropical disease.

## Figures and Tables

**Table 1:** Solubility values of praziquantel (PZQ), physical mixture (PM) of PZQ-GCC and interaction product (IP) of PZQ-GCC in acid and simulated intestinal fluid (SIF) media (mean values ± standard deviation; *n* = 0.07).

Sample	HCl 0.001 M Medium, pH = 3	SIF Medium, pH = 6.8
PZQ	0.50 mg/mL	0.45 mg/mL
PM PZQ-GCC	0.52 mg/mL	0.50 mg/mL
IP PZQ-GCC	1.42 mg/mL	0.73 mg/mL

## Supplementary Materials

The following are available online at https://www.mdpi.com/1999-4923/11/10/533/s1, Section 1: Solid State Characterization of Calcium Carbonate/Praziquantel Systems. Figure S1: Powder X-ray diffraction (XRD) patterns of praziquantel (PZQ), ground calcium carbonate (GCC), physical mixture (PM) of PZQ-GCC and interaction product (IP) of PZQ-GCC. Figure S2: DSC curves of PZQ, GCC, PM and IP. Figure S3: TGA profiles of the GCC, IP, PM, and PZQ solids, Figure S4: SEM micropictures of GCC, pristine PZQ, PM and IP. Figure S5: FTIR (a), and Raman (b) spectra of PZQ, GCC, IP and PM.
