# Supplementary material for: Ground Calcium Carbonate as a Low Cost and Biosafety Excipient for Solubility and Dissolution Improvement of Praziquantel
Source: Pharmaceutics. 2019 Oct 14;11(10):533. doi: 10.3390/pharmaceutics11100533 (PMC6835254; doi:10.3390/pharmaceutics11100533)
Supplement: Supplementary file 1 [file pharmaceutics-11-00533-s001.pdf]

pharmaceutics

MDPI

Article

Supplementary Materials: Ground Calcium Carbonate as a Low Cost and Biosafety Excipient for Solubility and Dissolution Improvement of Praziquantel

Ana Borrego-Sánchez, Rita Sánchez-Espejo, Beatrice Albertini, Nadia Passerini, Pilar Cerezo, César Viseras, and C. Ignacio Sainz-Díaz

1. Solid State Characterization of Calcium Carbonate/Praziquantel Systems

Powder XRD diffractograms of PZQ, GCC, and the physical mixture (PM) and the interaction product (IP) of both solids (PZQ and GCC) are shown in Figure S1. PZQ shows the most intense reflection around 16.5° (2θ units) and other intense reflections at approximately 8°, 19.1° and 23.3° (2θ units) according with that previously reported [1]. GCC shows a typical pattern of calcite with reflections at 23.0°, 29.4° (the most intense), 39.5° and 43.1° (2θ units). The XRD pattern of IP was previously reported [2], nevertheless it has been included in this work for comparison purposes. The solid IP showed only reflections of calcium carbonate crystals, apparently due to the amorphization of PZQ or a partial reduction of crystallinity of PZQ. Previous works reported a similar loss of crystallinity of PZQ through the formation of a solid dispersion with different excipients [3–5]. On the contrary, the PZQ and GCC PM showed reflections of both components though those of PZQ have low intensity due to its low proportion in the mixture.

DSC analysis of these samples clearly shows some modification of the IP solid state. In particular, the DSC curve of IP (Figure S2) displayed a broad peaks at 110–112 °C and 133–136 °C and a shoulder around 142–144 °C, indicating an alteration of the crystal structure of the pristine PZQ [2,4,6]. On the contrary, both PZQ and PM showed a strong endothermic peak at 142–144 °C corresponding to the melting point of the (RS)-PZQ crystal, while GCC did not present thermal events in the considered range of temperature.

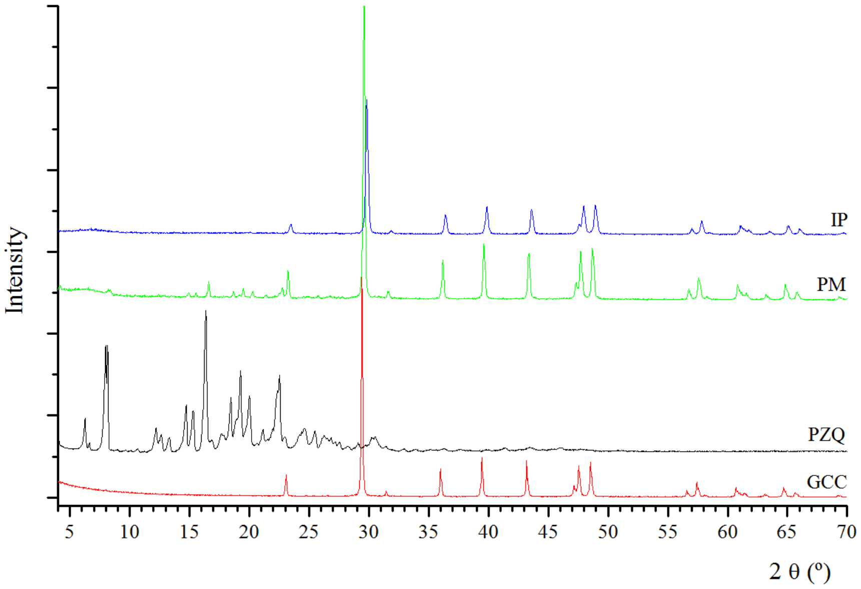

Figure S1. Powder X-ray diffraction (XRD) patterns of praziquantel (PZQ), ground calcium carbonate (GCC), physical mixture (PM) of PZQ-GCC and interaction product (IP) of PZQ-GCC.

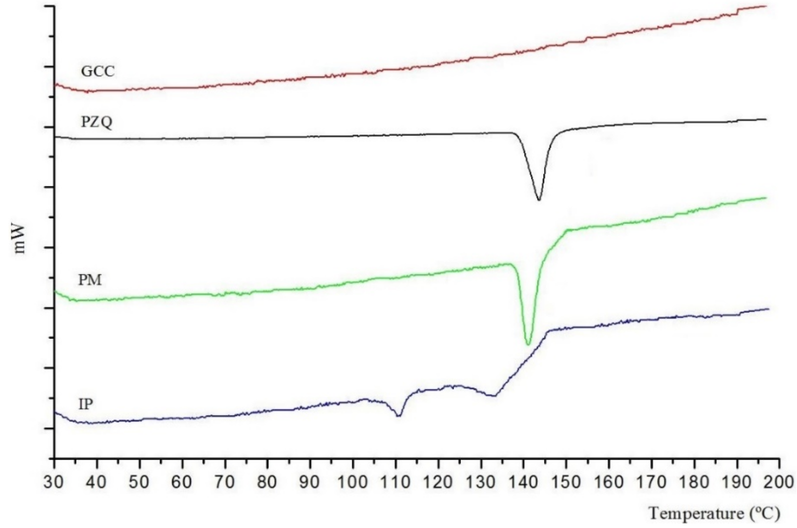

Figure S2. DSC curves of PZQ, GCC, PM and IP.

Thermogravimetric profiles of the samples studied are shown in Figure S3. GCC shows a slight weight loss (0.34% *w/w*) at 40 °C approximately, corresponding to the surface humidity evaporation. PZQ weight loss occurs in the range between 200 and 422 °C (97.64% *w/w*) due to the decomposition of PZQ. The PM showed a similar profile as that of the IP solid [2] with the decomposition of the PZQ fraction at 250–300 °C.

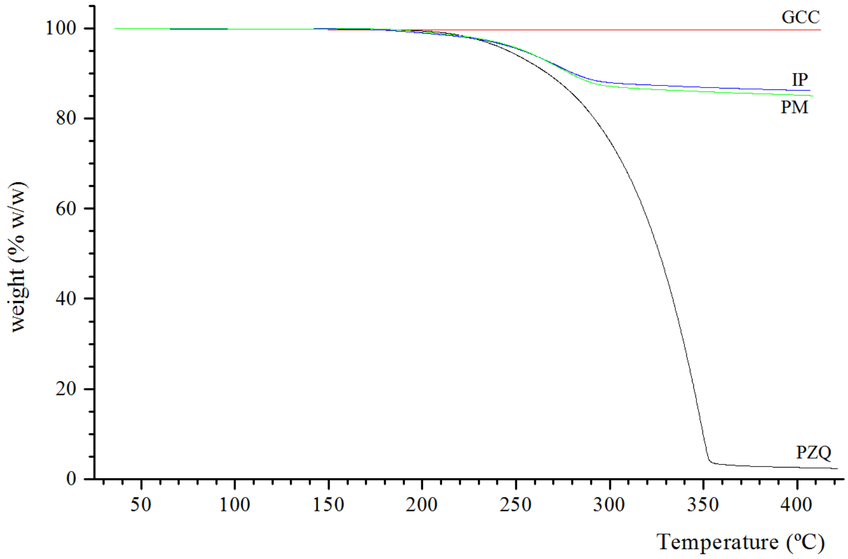

Figure S3. TGA profiles of the GCC, IP, PM, and PZQ solids.

SEM micro picture of GCC shows particle size lower than 10 μm in the (Figure S4A) with typical morphologies of high purity calcium carbonate for pharmaceutical use [7]. Microphotographs of the pristine racemic (RS)-PZQ presented elongated prismatic morphology with sizes > 100 μm (Figure S4B), similar to that observed in previous racemic PZQ mixtures [2,3,8,9].

SEM microphotographs of PM clearly show particles of both components, (RS)-PZQ and GCC without appreciable changes in morphology, texture or particle size (Figure S4C and S4D), whereas SEM pictures of IP highlight important changes of texture and morphology (Figure S4E and S4F). The initial structure of PZQ disappeared and fibrous and filamentous particles of PZQ surrounding the GCC particles were observed. The size reduction of PZQ may be a further explanation of the absence of reflection peaks in the IP XRD pattern, as reported in previous works [3–5,10]. In fact, the particle size of pristine PZQ was in the range 40–550 μm, with a sample particle size such that 50% of the

mass of the particles is of a smaller diameter and 50% of the particles is of a larger diameter d50 = 140.5 µm. GCC particles were around 1–28 µm with a d50 = 7.1 µm. The PM showed a particle size distribution in the range 200–1000 µm with a d50 = 457.9 µm, indicating that when the physical mixture is prepared, aggregates of particles are formed that produce an increase in the particle size with respect to the pure samples. Moreover, the particle size of the IP was smaller than that of the PM PZQ-GCC, being around 80–550 µm with a d50 = 217.6 µm.

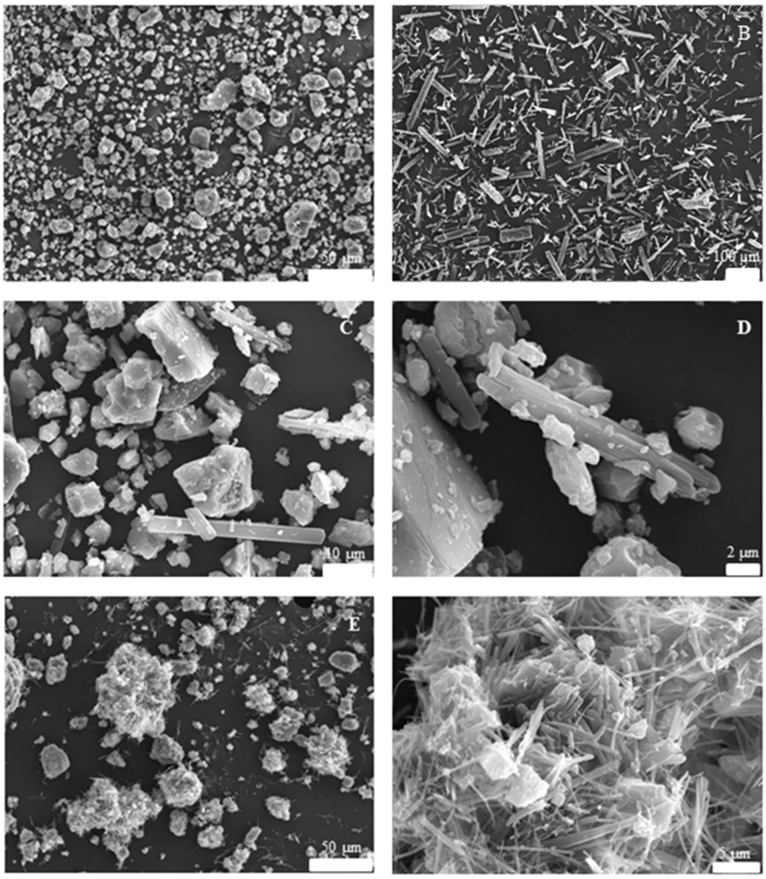

Figure S4. SEM micropictures of GCC, pristine PZQ, PM and IP.

Spectroscopical properties of GCC, pristine PZQ, PM and IP were compared with FTIR (Figure S5a) and Raman (Figure S5b) spectra. GCC shows characteristic IR bands of the carbonate group (CO_3^{2-}) at 1453, 875 and 712 cm^{-1} [11,12]. The IR spectrum of the pristine PZQ shows two bands in the range 2960–2840 cm^{-1} that were assigned to stretching ν(CH) modes of the alkyl CH and CH_2 groups [3,10,13]. Two ν(C=O) bands were observed at 1665–1621 cm^{-1} [3,10,13], which we assigned to each carbonyl group of PZQ with different local environments in the racemic crystal packing. The *δ*(C–H) and *ν*(C–N) modes are observed around 1350–1021 cm^{-1} [14]. In the PM and IP the bands of PZQ were detected but no big differences could be observed due to the high proportion of GCC, whose bands overlap those of PZQ that is in low relative proportion in the PM mixture. Nevertheless, slight differences are detected in the ν(C=O) bands. They are closer in IP than in PM and pristine PZQ. The Raman spectrum of PZQ (Figure S5b) shows a band at 1044 cm^{-1} that we assigned to a symmetric $\delta(CN)_s$ vibration mode of the C-N bonds. On the other hand, a high intensity band appears at 1085 cm^{-1} in the GCC spectrum that we assigned to a symmetric ν(C–O) stretching mode of the carbonate C–O bond [15–17]. The PM and IP mixtures showed bands of PZQ and GCC. In PM, the PZQ component showed the same bands in the same frequencies and relative intensity that as isolated PZQ solid in the pristine racemic (RS)-PZQ crystals. However, the IP mixture showed slight differences in relative intensities and frequencies of the bands with respect to pristine PZQ according to previous results [10].

(**a**)

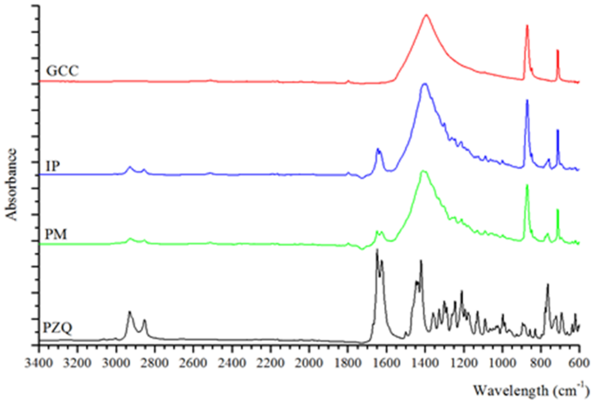

(**b**)

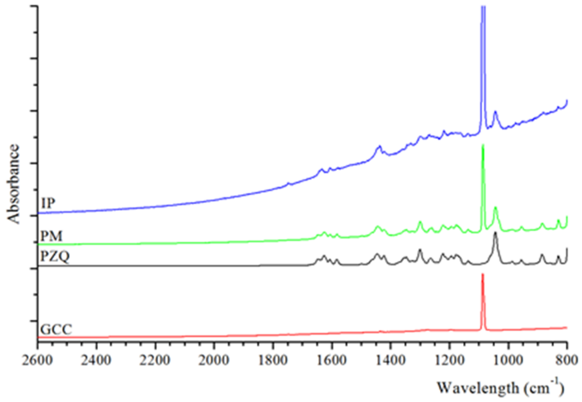

Figure S5. FTIR (**a**), and Raman (**b**) spectra of PZQ, GCC, IP and PM.

References

1. Lindenberg, M.; Kopp, S.; Dressman, J.B. Classification of orally administered drugs on the World Health Organization Model list of Essential Medicines according to the biopharmaceutics classification system. *Eur. J. Pharm. Biopharm*. **2004**, *58*, 265–278.
2. Perissutti, B.; Passerini, N.; Trastullo, R.; Keiser, J.; Zanolla, D.; Zingone, G.; Voinovich, D.; Albertini, B. An explorative analysis of process and formulation variables affecting comilling in a vibrational mill: The case of praziquantel. *Int. J. Pharm*. **2017**, *533*, 402–412.
3. Mourao, S.C.; Costa, P.I.; Salgado, H.R.N.; Gremiao, M.P.D. Improvement of antischistosomal activity of praziquantel by incorporation into phosphatidylcholine-containing liposomes. *Int. J. Pharm*. **2005**, *295*, 157–162.
4. Becket, G.; Schep, L.J.; Tan, M.Y. Improvement of the in vitro dissolution of praziquantel by complexation with α-, β-, and γ-cyclodextrins. *Int. J. Pharm*. **1999**, *179*, 65–71.
5. Cheng, L.; Lei, L.; Guo, S. In vitro and in vivo evaluation of praziquantel loaded implants based on PEG/PCL blends. *Int. J. Pharm*. **2010**, *387*, 129–138.
6. Zanolla, D.; Perissutti, B.; Passerini, N.; Chierotti, M.R.; Hasa, D.; Voinovich, D.; Gigli, L.; Demitri, N.; Geremia, S.; Keiser, J.; et al. A new soluble and bioactive polymorph of praziquantel. *Eur. J. Pharm*. Biopharm. **2018**, *127*, 19–28.
7. Rowe, R.C.; Sheskey, P.J.; Weller, P.J. Calcium Carbonate. In: *Handbook of Pharmaceutical Excipients*, 6th ed.; Pharmaceutical Press: London, UK, 2009.
8. Liu, Y.; Wang, X.; Wang, J.K.; Ching, C.B. Structural Characterization and Enantioseparation of the Chiral Compound Praziquantel. *J. Pharm. Sci*. **2004**, *93*, 3039–3046.
9. Espinosa-Lara, J.C.; Guzmán-Villanueva, D.; Arenas-García, J.I.; Herrera-Ruiz, D.; Rivera-Islas, J.; Román-Bravo, P.; Morales-Rojas, H.; Höpfl, H. Cocrystals of active pharmaceuticals ingredients-praziquantel in

combination with oxalic, malonic, succinic, maleic, fumaric, glutaric, adipic and pimelic acids. *Cryst. Growth Des*. **2013**, *13*, 169–185.

10. Borrego-Sánchez, A.; Hernández-Laguna, A.; Sainz-Díaz, C.I. Molecular modeling and infrared and Raman spectroscopy of the crystal structure of the chiral anti-parasitic drug praziquantel. *J. Mol. Model*. **2017**, *23*, 106.
11. Reig, F.B.; Adelantado, J.V.G.; Moreno, M.C.M.M. FTIR quantitative analysis of calcium carbonate (calcite) and silica (quartz) mixtures using the constant ratio method. Application to geological samples. *Talanta* **2002**, *58*, 811–821.
12. Vagenas, N.V.; Gatsouli, A.; Kontoyannis, C.G. Quantitative analysis of synthetic calcium carbonate polymorphs using FT-IR spectroscopy. *Talanta* **2003**, *59*, 831–836.
13. Borrego-Sánchez, A.; Viseras, C.; Aguzzi, C.; Sainz-Díaz, C.I. Molecular and crystal structure of praziquantel. Spectroscopic properties and crystal polymorphism. Eur. J. Pharm. Sci. **2016**, *92*, 266–275.
14. Borrego-Sánchez, A.; Carazo, E.; Aguzzi, C.; Viseras, C.; Sainz-Díaz, C.I. Biopharmaceutical improvement of praziquantel by interaction with montmorillonite and sepiolite. *Appl. Clay Sci*. **2018**, *160*, 173–179.
15. Edwards, H.G.M.; Villar, S.E.J.; Jehlicka, J.; Munshi, T. FT–Raman spectroscopic study of calcium-rich and magnesium-rich carbonate minerals. *Spectrochim. Acta A* **2005**, *61*, 2273–2280.
16. De Paula, S.M.; Huila, M.F.G.; Araki, K.; Toma, H.E. Confocal Raman and electronic microscopy studies on the topotactic conversion of calcium carbonate from Pomacea lineate shells into hydroxyapatite bioceramic materials in phosphate media. *Micron* **2010**, *41*, 983–989.
17. Smith, G.P.S.; Gordon, K.C.; Holroyd, S.E. Raman spectroscopic quantification of calcium carbonate in spiked milk powder samples. *Vib. Spectrosc*. **2013**, *67*, 87–91.

© 2019 by the authors. Submitted for possible open access publication under the terms and conditions of the Creative Commons Attribution (CC BY) license (http://creativecommons.org/licenses/by/4.0/).
